# A Rational Approach to Understanding and Evaluating Responsive Neurostimulation

**DOI:** 10.1007/s12021-019-09446-7

**Published:** 2020-01-09

**Authors:** Nathaniel D. Sisterson, Thomas A. Wozny, Vasileios Kokkinos, Anto Bagic, Alexandra P. Urban, R. Mark Richardson

**Affiliations:** 1 Department of Neurological Surgery, Brain Modulation Lab, University of Pittsburgh, Pittsburgh, PA USA; 2 Department of Neurological Surgery, University of California, San Francisco, San Francisco, CA USA; 3 Department of Neurological Surgery, Massachusetts General Hospital, Boston, MA USA; 4 Harvard Medical School, Boston, MA USA; 5 Department of Neurology, University of Pittsburgh, Pittsburgh, PA USA; 6 University of Pittsburgh Comprehensive Epilepsy Center, Pittsburgh, PA USA

**Keywords:** Closed-loop, Neuromodulation, Seizure detection, Device configuration, Extrapolation, Drug-resistant epilepsy

## Abstract

Closed-loop brain stimulation is increasingly used in level 4 epilepsy centers without an understanding of how the device behaves on a daily basis. This lack of insight is a barrier to improving closed-loop therapy and ultimately understanding why some patients never achieve seizure reduction. We aimed to quantify the accuracy of closed-loop seizure detection and stimulation on the RNS device through extrapolating information derived from manually reviewed ECoG recordings and comprehensive device logging information. RNS System event logging data were obtained, reviewed, and analyzed using a custom-built software package. A weighted-means methodology was developed to adjust for bias and incompleteness in event logs and evaluated using Bland–Altman plots and Wilcoxon signed-rank tests to compare adjusted and non-weighted (standard method) results. Twelve patients implanted for a mean of 21.5 (interquartile range 13.5–31) months were reviewed. The mean seizure frequency reduction post-RNS implantation was 40.1% (interquartile range 0–96.2%). Three primary levels of event logging granularity were identified (ECoG recordings: 3.0% complete (interquartile range 0.3–1.8%); Event Lists: 72.9% complete (interquartile range 44.7–99.8%); Activity Logs: 100% complete; completeness measured with respect to Activity Logs). Bland–Altman interpretation confirmed non-equivalence with unpredictable differences in both magnitude and direction. Wilcoxon signed rank tests demonstrated significant (*p* < 10^−6^) differences in accuracy, sensitivity, and specificity at >5% absolute mean difference for extrapolated versus standard results. Device behavior logged by the RNS System should be used in conjunction with careful review of stored ECoG data to extrapolate metrics for detector performance and stimulation.

**Electronic supplementary material:**

The online version of this article (10.1007/s12021-019-09446-7) contains supplementary material, which is available to authorized users.

## Introduction

As early as 1954, Penfield reported the modulatory effects of electrical stimulation on seizures, observed by electrocorticography (ECoG) (Penfield and Jasper 1954). Based on this and subsequent observation (Durand 1986; Kinoshita et al. 2004, 2005; Kossoff et al. 2004), the NeuroPace RNS System was developed as a closed-loop brain modulation device capable of detecting and responding to abnormal brain activity by delivering programmable stimulation targeted to seizure foci, with the intention of disrupting epileptiform activity before a seizure can develop (Heck et al. 2014). The ability of the device to respond to abnormal brain activity is contingent upon the degree to which the physician-selected combination of detection settings are suited for the particular seizure onset pattern(s) observed in ECoG recordings for each patient (Sun et al. 2008).

The efficacy of the RNS System has been demonstrated by several multi-center outcomes studies, in which a median 55% of patients experienced a 53% reduction in seizure frequency at 2 years and a median 70% of patients experienced a 78% seizure frequency reduction at 6 years (Bergey et al. 2015; Geller et al. 2017; Jobst et al. 2017). However, patients remain at increased risk for severe complications, such as severe injuries and sudden unexplained death (SUDEP), during the interval to seizure control (Banerjee et al. 2009). The increase in responder rate and seizure reduction over time raises the question of why some patients achieve a faster and greater response.

One approach to this question is to explore the characteristics of patients who are responders versus non-responders. This approach, however, is insufficient to disentangle whether poor patient response is due to suboptimal settings or intrinsic patient characteristics, because it is severely confounded by the heterogeneity of device behavior between patients. This heterogeneity results from the interaction between the detection and stimulation settings and an individual patient’s distinct neurophysiological seizure onset patterns. Thus, we propose that the first step to improving closed-loop therapy is to develop quantitative methods for evaluating device behavior, defined by the type of neurophysiological event being stimulated, the timing of stimulation relative to event onset, and the rate of stimulation. This strategy extends significantly beyond the scope of current analysis tools available via the NeuroPace Patient Data Management System (PDMS).

Here, we report our comprehensive analysis of logging information generated by the RNS System. Based on findings of incomplete or overwritten information about detections and stimulations and reporting bias, we hypothesized that extrapolation of information derived from manually reviewed ECoG recordings using comprehensive aggregate logging information (which by itself lacks details about specific detections and stimulations) would reveal a clinically significant and unpredictable difference in device behavior, as compared to current, standard methods available in the PDMS (S1 Table). Finally, we report the results of the extrapolation method in the context of clinical outcomes, to highlight the heterogeneity of device behavior between patients.

## Material and Methods

### Study Design and Cohort Selection

We performed a retrospective chart review approved by the University of Pittsburgh Institutional Review Board (IRB). First, we collected RNS System device data and performed an in-depth analysis of the logging capabilities. Second, we used the information generated by this analysis to perform a quantitative evaluation of device behavior (detections and stimulations).

All subjects underwent intracranial monitoring via stereo-electroencephalography (SEEG), prior to RNS therapy (RNS, NeuroPace, Mountain View, CA, USA), as recommended by a multidisciplinary epilepsy surgery team. RNS implantations occurred consecutively between January 2015 and November 2017. All related clinical data were abstracted from the electronic medical records systems or obtained by patient surveys. ECoG recordings and associated metadata generated by the RNS System were obtained from the PDMS (accessed between November 2016 and December 2017).

### Implantation and Programming

RNS electrodes were targeted to epileptogenic zones identified during intracranial monitoring via SEEG. No neural stimulation was delivered for approximately one month after implantation, in order to observe baseline brain activity and detector performance. Patients, or their caregivers, were also asked to keep a seizure diary to log the time, duration, and manifestations of seizures. Once the clinical team reached consensus on the presumed acceptable accuracy for the detection parameters, initial stimulation settings were configured and enabled so that the device delivered detection-triggered stimulation therapy.

The RNS System parameter space can be separated into two main categories: detection parameters and stimulation parameters (S1 Fig). The three primary types of detectors are bandpass, line length, and area, with additional built-in detectors for saturation and noise. Two channels are selected to be detection channels for a total of two detecting electrode pairs. Next, up to two first-order Patterns, referred to as Pattern A and Pattern B are configured. Each Pattern can be further comprised of up to two second-order patterns, referred to as Pattern A1, Pattern A2, Pattern B1, and Pattern B2. Each of these second-order Patterns corresponds to a single detection channel and detector. Stimulation can occur from all eight electrodes, and settings are configured for up to five consecutive discretely triggered stimulations, referred to as therapies, comprised of up to two consecutive bursts each. The cathode (positive) and anode (negative) electrode montage and current affect the volume of tissue treated and must be configured for each burst. The bursts of the first therapy only may be configured to respond differently to specific Patterns. The total number of therapies in a given 24-h window are also limited by a programmable amount.

On an approximately monthly basis, the clinical team reviewed the information recorded by the device, as well as patient reported outcomes. The team decided whether, and how, to adjust the device detection or stimulation parameters based on a combination of summary data obtained from the standard PDMS interface and clinical interviews. The period of time during which detection and stimulation settings remain consistent is referred to as a programming epoch. The primary factors driving RNS adjustments were the approximate daily number of events and the number of long episodes and saturations since the most recent programming epoch. This process represents the current, or standard, method for evaluating device behavior.

### Data Acquisition

**Fig. 1:**
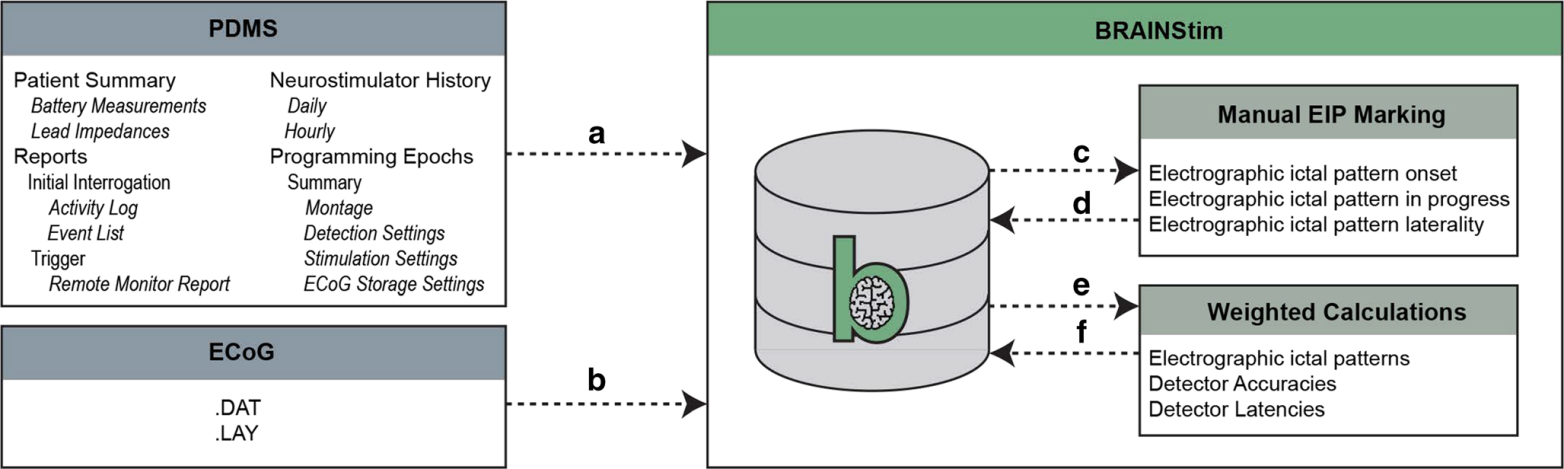
Flow diagram of data loading, pre-processing, manual review, and calculations using the BRAINStim© platform. **a** Data crucial to the analysis of RNS System performance are loaded from the PDMS using a custom C#.NET HTML parsing tool. **b** Raw ECoG data, along with hardware diagnostic information, are loaded from files provided by NeuroPace. **c** ECoG data are sorted into groups by programming epoch, which are exported as .EDF files. **d** ECoG data are manually reviewed, and EIP onset and laterality are annotated. All data are imported back into the database and merged with the original files. **e** Weighted calculation scripts are executed on the database. **f** The results of the scripts are loaded back into the database and used to generate figures, as well as to facilitate further analysis

We obtained ECoG recordings from NeuroPace via an encrypted USB drive on a quarterly basis. To obtain the majority of the data needed for analysis, including device detection and stimulation settings, event timestamps, total number of events per interrogation, and summary logging data, we created a custom HTML parsing tool in C#. NET to programmatically load and transform data from the PDMS into our local database on a weekly basis (Fig. 1). All data were loaded in a SQL Server (Microsoft, Redmond, Washington, USA) database using extract, transform, and load (ETL) code developed for SQL Server Integration Services. The database was secured by schema, and the primary data set was de-identified using an automated process. Patient identifiers were stored in a separately secured database. This database and associated software used in our subsequent analyses form the basis of the BRAINStim© (Biophysically Rational Analysis for Informed Neural Stimulation) platform.

### Logging Analysis

We reviewed the different levels of logging available on the PDMS, including *ECoG Reports*, *Event List*, *Activity Log*, and *Neurostimulator History*. *ECoG Reports*, *Event List*, and *Activity Log* are complementary log summaries of each interrogation while the *Neurostimulator History* provides a histogram summarizing the same information, separately located on the PDMS. We assessed the level of detail available and calculated the amount of data missing from each level of logging, using the *Activity Log* as the source of truth for completeness for interrogation data (*ECoG Reports*, *Event List*, and *Activity Log*), and the histogram_data_missing and diagnostic_data_missing flags for the daily and hourly *Neurostimulator History*.

### Device Behavior Analysis

To evaluate device behavior, we looked at whether or not the neurophysiological event being stimulated was an electrographic ictal pattern (EIP). In this study, EIPs represent probable seizure events, but we use this term since the confirmation of a seizure event requires additional clinical evidence not available through the RNS System. First, we reviewed ECoG recordings and calculated sensitivity, specificity, and accuracy of the detectors with respect to EIPs by extrapolating this information using logging data. Next, we calculated latency to evaluate the timing of stimulation relative to seizure onset. To look at rate of stimulation, we calculated the mean number of stimulations delivered per day. We tested our hypothesis, that the weighted method would reveal a clinically significant difference in device behavior as compared to the standard, non-weighted method, using Bland-Altman plots and Wilcoxon signed rank tests. Finally, we calculated the mean, median, interquartile range, and range of device behaviors generated by the extrapolated calculations to demonstrate the heterogeneity of device behavior across patients.

All ECoG recordings were reviewed by an experienced neurophysiologist (VK) to identify the presence or absence of an EIP. True positive, false positive, true negative, and false negative rates were calculated for each episode from the manually reviewed ECoG data using the initial second-order *Pattern* (e.g. *Pattern A1*, *Pattern A2*, etc.) to trigger (S2 Fig). Each second-order *Pattern* was further grouped by episode length (episode versus long episode). To perform weighted calculations, standard ECoG calculations were weighted using the percentage of each trigger (e.g. *Pattern A1*, *Pattern A2*, etc.) and pattern type (e.g. episode, long episode), as reported by the daily *Neurostimulator History*. As shown below, *Pattern* accuracy for all detectors stratified by episodes (*A*1 _ *E*_*ECoGAcc*_) and long episodes (*A*1 _ *LE*_*ECoGAcc*_) were calculated first. Next, the weights were calculated using the percentage of times a given *Pattern* triggered an episode multiplied by (*A*1 _ *E*_*ECoG*_) the percentage of *Patterns* for the entire epoch (*A*1_*History*_). Long episode weights were calculated by multiplying the percentage of times a given *Pattern* triggered a long episode by the percentage of long episodes for the entire epoch (*LE*_*History*_). Finally, the weights were applied to each detector stratified by episode versus long episode and summed, resulting in the weighted accuracy for the programming epoch (*PE*_*Acc*_).

$$ A1\_{E}_{ECo{G}_{Acc}}=\left(A1\_{E}_{ECo{G}_{TP}}+A1\_{E}_{ECo{G}_{TN}}\right)/\left(A1\_{E}_{ECo{G}_{TP}}+A1\_{E}_{ECo{G}_{TN}}+B1\_{E}_{ECo{G}_{TP}}+B2\_{E}_{ECo{G}_{TN}}\right) $$

$$ A1\_{E_{WT}}_{History}=A1\_{E}_{ECoG}/\left(A1\_{E}_{ECoG}+A2\_{E}_{ECoG}+B1\_{E}_{ECoG}+B2\_{E}_{ECoG}\right)\times A{1}_{History}/\left(A{1}_{History}+A{2}_{History}+B{1}_{History}+B{2}_{History}\right) $$

$$ A1\_L{E_{WT}}_{History}=A1\_{LE}_{ECoG}/\left(A1\_{LE}_{ECoG}+A2\_{LE}_{ECoG}+B1\_{LE}_{ECoG}+B2\_{LE}_{ECoG}\right)\times L{E}_{History}/\left(L{E}_{History}+{E}_{History}\right) $$

$$ P{E}_{Acc}=A1\_{E_{ECoG}}_{Acc}\times A1\_{E_{WT}}_{History}+A2\_{E_{ECoG}}_{Acc}\times A2\_{E_{WT}}_{History}+B1\_{E_{ECoG}}_{Acc}\times B1\_{E_{WT}}_{History}+B2\_{E_{ECoG}}_{Acc}\times B2\_{E_{WT}}_{History}+A1\_{LE_{ECoG}}_{Acc}\times A1\_L{E_{WT}}_{History}+A2\_{LE_{ECoG}}_{Acc}\times A2\_{LE_{WT}}_{History}+B1\_{LE_{ECoG}}_{Acc}\times B1\_L{E_{WT}}_{History}+B2\_L{E_{ECoG}}_{Acc}\times B2\_L{E_{WT}}_{History} $$

We repeated this weighted-mean methodology to calculate detector latency, defined as the number of seconds elapsed between the manually marked EIP onset and the first detection event of the first episode of a given ECoG recording. Only recordings in which the RNS System correctly identified an EIP (true positive) were used, and recordings beginning with an in-progress episode were excluded. The weighted mean paradigm was similarly used for each patient to calculate mean number of stimulations per episode and EIPs per month. The mean pattern detection rate per hour, total events per hour, percentage of days reaching the daily therapy limit, number of programming epochs, and time to enabling stimulation were calculated directly from log data (no weighting was necessary).

The number of ECoG recordings stored on the device at any given time is limited to approximate four 90-s 4-channel recordings, which represent only a subset of all recorded events due to constant overwriting of data. To determine the extent to which stored ECoGs may adequately reflect true overall device behavior, we applied a weighted mean methodology to ECoG-dependent calculations (detector sensitivity, specificity, accuracy, number of EIPs, and number of therapies) and compared these results to those obtained using the standard (non-weighted) PDMS methodology using Bland–Altman plots (Bland and Altman 1986; Giavarina 2015). To test our hypothesis, we performed Wilcoxon signed rank tests of extrapolated versus standard calculations for the accuracy, sensitivity, and specificity of each patient programming epoch, with clinical significance set to >5%.

We quantified heterogeneity of device behavior between patients by calculating the mean, median, 25th and 75th percentiles, and range of the accuracy, sensitivity, specificity and latency obtained by the extrapolated calculations. We additionally calculated the total number of stimulations delivered to the left versus right hemisphere at 8.5 months post-implantation for patients with bilateral lead placements, again using the extrapolated results.

### Clinical Data Acquisition and Analysis

We performed a retrospective review of the UPMC electronic medical record to obtain medical history, surgical history, seizure classifications, anti-epileptic drug regimens, and natural course of disease. Due to the incomplete and inconsistent documentation of seizure activity, we evaluated clinical outcomes using two complementary patient reported outcomes surveys. To measure quality of life outcomes, we used the Personal Impact of Epilepsy Scale (PIES) instrument, a compact 25 question survey which detects changes in impact of seizures, medication side effects, impact of comorbidities, and overall quality of life (Fisher et al. 2015). To measure clinical seizure outcomes, we used as a custom questionnaire to document seizure type and frequency, duration, intensity, and loss of consciousness, as well as seizure diary and magnet swipe compliance (S3 Fig) (Sun and Morrell 2014). These questionnaires were administered to RNS patients at their most recent clinic visit, at which time they reported both pre- and post-RNS outcomes.

We calculated the mean, median, interquartile range, and range for age at time of implant, years of uncontrolled seizures, months implanted with the RNS System, and number of failed anti-seizure drugs (ASDs). Quality of life was quantified using the PIES instrument. Both the percent and absolute improvement in pre- and post-RNS survey scores were calculated and compared for each patient to determine whether quality of life improved, declined, or remained the same. Both the percent and absolute change in seizure frequency, duration, and severity pre- and post-RNS survey results were compared for each patient. Finally, we used Pearson correlation to quantify the degree to which EIPs correlate with patient reported seizures for a corresponding one month period. All statistical analyses were performed using MATLAB (MathWorks, Natic, MA, USA) and R (R Core Team, Vienna, Austria).

## Results

### Demographics and Adverse Events

Twelve patients were included in this analysis, with a mean responsive stimulation therapy duration of 21.5 months (S2 Table; median 23.5; interquartile range 13.5–31; range 5–34). Five out of 12 patients exhibited epilepsy of structural etiology, as defined by the ILAE classification system (S3 Table) (Berg et al. 2010; Fisher 2017). A total of 14,394 ECoG files were processed, encompassing a total of 198.9 RNS-implanted months. Analysis revealed a total number of 4827 EIPs, with a mean of 71 EIPs per programming epoch (median 24; interquartile range 6.5–98; range 1–780). One patient underwent resection and electrode repositioning 366 days after RNS-recorded ECoG revealed primarily unilateral seizure onset, and subsequent data were excluded from our analyses. Another patient experienced throbbing headaches post-implantation that resolved within one month. There were no other adverse events recorded and no surgical infections.

### Logging Results

**Fig. 2:**
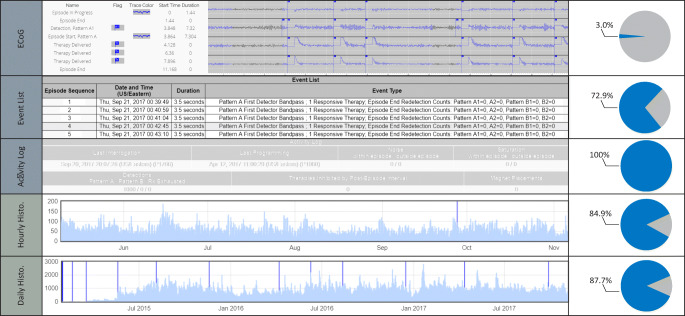
Levels of RNS System detailed and summary logging. Completeness of each source of logging information relative to the Activity Log, which is the most complete but least detailed logging source, is shown in pie charts. Representative screen captures of the NeuroPace PDMS provide a basic reference of the primary sources of logging data used to perform our analyses. Logging data (top) comes from the Reports tab for ECoG recordings (most detailed) and the interrogation *Event List* and *Activity Log* (most complete); additional summary data (bottom) comes from the *Neurostimulator History* tab for daily and hourly histogram data

We found that the RNS System is limited in the granularity of information reliably captured and made available via the PDMS (Fig. 2). The richest source of information comes from stored ECoG recordings, which includes up to 4 channels of ECoG and corresponding event timestamps identifying second-order *Patterns* and therapies. Because the onboard storage space available for ECoG recordings on the device is extremely limited, only a small subset of recordings is preserved relative to the continuous neural signal analyzed online by the device. The mean percentage of triggered ECoG recording files uploaded to the PDMS per programming epoch, relative to total events (defined as the sum of pattern detection, saturation, and magnet swipe events), was 3.0% (median 0.6%; interquartile range 0.3–1.8%; range 0–39.8%). The remainder of the files were either not configured for storage and/or overwritten due device storage constraints.

The next most detailed source of information comes from the *Event List*, which includes the episode onset timestamp and a summary of the second-order *Patterns* and therapies that occurred. The *Event List* can hold up to approximately 700 events between interrogations, after which storage capacity is reached and additional new events are lost. Relative to total events, the *Event Lists* were 72.9% (median 80.0%; interquartile range 44.7–99.8%; range 16.2–100%) complete in our cohort. Finally, the *Activity Log* contains summary data for first-order *Patterns* during the time period since the previous interrogation. The mean number of hours between interrogations was 30.3 (median 24; interquartile range 23–26; range 0–2583). The storage available for this information is sufficient such that the device is unlikely to ever lose any summary data. Separately, the *Neurostimulator History* contains daily summary data going back up to 10 years and hourly summary data going back up to 180 days. Summary data is overwritten at 255 events in a one-hour period. Relative to total events, the *Neurostimulator History* was 84.9% (median 100%, interquartile range 72.0–100%, range 37.8–100%) complete in our cohort.

### Device Behavior Results

The mean time to enabled neural stimulation therapy was 45.9 days (median 37 days, interquartile range 29–66.75 days, range 8–90 days). The mean number of programming events was 3.7 per year per patient (median 3.5, interquartile range 2.8–4.3, range 1.8–6). Responsive stimulation activity was characterized by a mean of 39.5 events per hour per patient (median 26.6; interquartile range 8.7–51.2; range 0–244.5). The mean number of stimulations per episode per patient was 1.1 (median 1.1, interquartile range 1.0–1.2, range 0–5), and the daily therapy limit (mean 2039.2, median 2000, interquartile range 1000–3000, range 1000–4000) was reached for 5.6% of the total treatment days (median 1.2%, interquartile range 0.2–8.6%, range 0–27.9%).

**Fig. 3:**
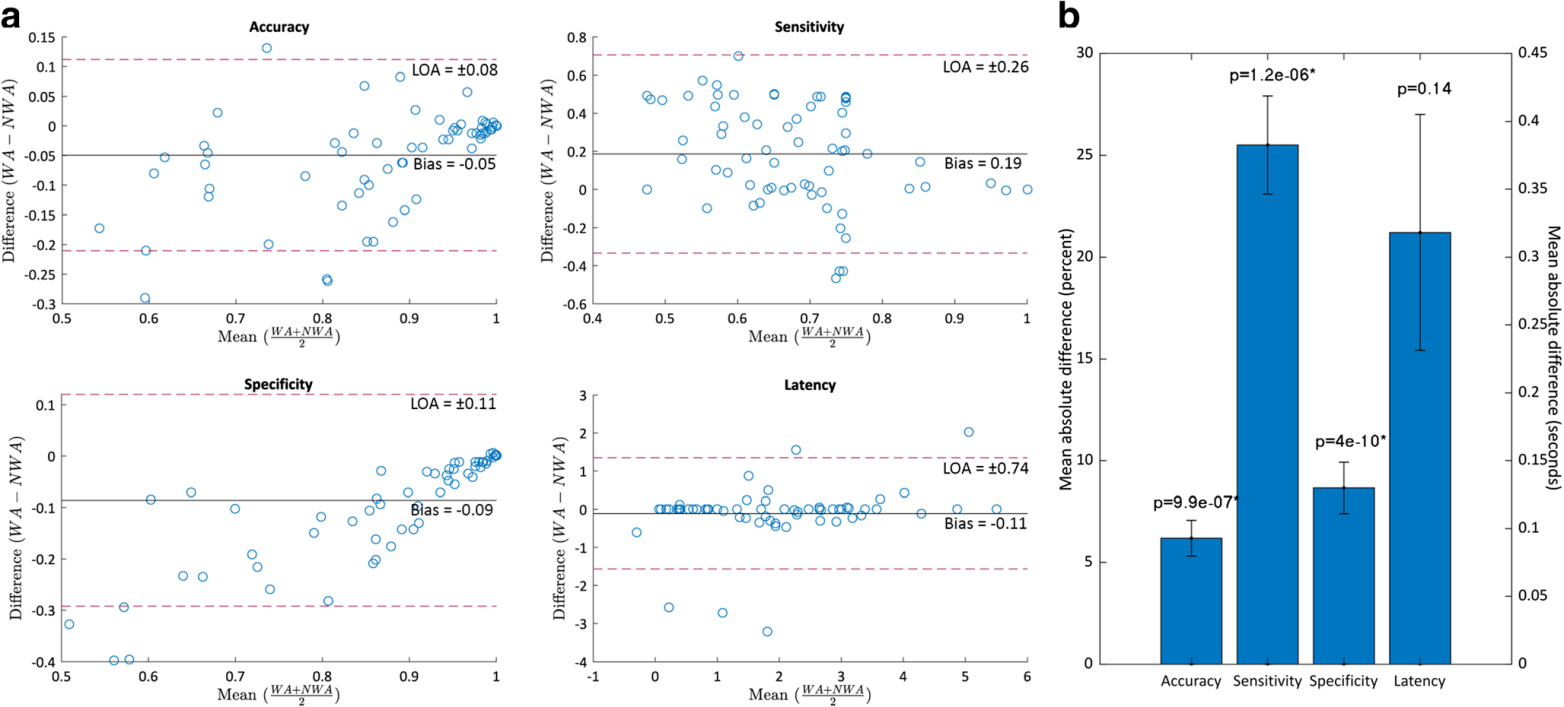
**a** Bland–Altman plots. For weighted accuracy, there was a negative bias with significant dispersion, and a slight positive trend between the mean and difference, with greater scatter as the mean decreases. For weighted latency, there was a negative bias with minimal dispersion, and some scattering at all values. For weighted sensitivity, there was a positive bias with significant dispersion and no clear trend, but with less scatter above a mean of 80%. For weighted specificity, there was a negative bias moderate dispersion from the bias with a positive trend between mean and difference, with greater scatter as the mean decreases. **b** Absolute mean difference of standard and weighted calculations. Accuracy, sensitivity, and specificity all have statistically, as well as clinically significant differences (defined as a difference of >5%) in extrapolated versus standard calculations. The difference between extrapolated and standard latency calculations was not statistically significant

To evaluate the difference between standard and extrapolated methods, we calculated both standard (S) means and means weighted by *Neurostimulator History* data (W), for detection accuracy (S = 90.0%, median 94.8%, interquartile range 84.3–98.8%, range 63.0–100%; W = 85.1%, median 89.7%, interquartile range 75.9–98.2%, range 45.0–100%), sensitivity (S = 51.5%, median 50.0%, interquartile range 48.7–50.1%, range 0–100%; W = 68.1%, median 74.9%, interquartile range 48.4–94.5%, range 0–100%), and specificity (S = 93.2%, median 96.7%, interquartile range 90.6–99.6%, range 64.5–100%; W = 84.6%, median 92.4%, interquartile range 75.9–99.6%, range 34.5–100%), for each epoch between programming changes (S4 Table). The weighted mean detector latency was negative for three of 71 total programming epochs evaluated (−1.073 s, −0.278 s, −0.596 s), indicating that detection and stimulation preceded seizure onset in these cases. We used Bland–Altman plots to evaluate the difference between standard and weighted average calculations, which revealed a bias that was unpredictable in both magnitude and direction, with limits of agreement ≥8% for all measurements (Fig. 3a). Wilcoxon signed rank tests were significant (*p* < 10^−6^) with >5% difference between extrapolated and standard methods for accuracy, sensitivity, and specificity (Fig. 3b).

**Fig. 4:**
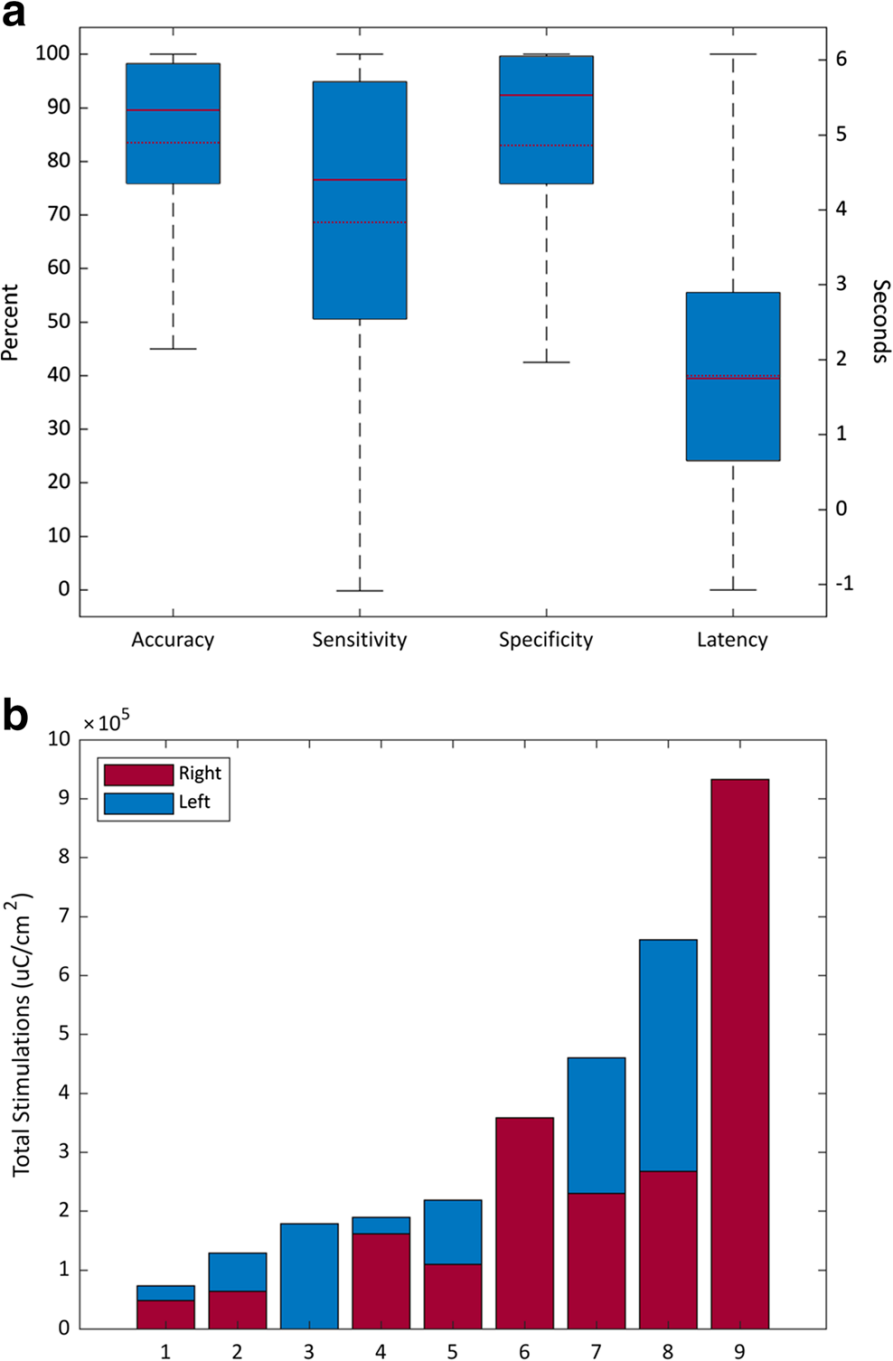
**a** Boxplots of mean, median, 25th and 75th percentiles, and range of accuracy, sensitivity, specificity, and latency. Wide interquartile ranges for accuracy, sensitivity, and specificity (*left axis*), with disagreement between the mean (dashed) and median (solid), revealing a heterogeneous and widely distributed differences in device behavior between patients. Latency (*right axis*) has a relatively narrower interquartile range, a wide range still exists. **b** Total stimulation per patient at 8.5 months post-implant. There is significant variability in the rate at which stimulation therapy is delivered between patients, with some patients receiving greater than 10 times the amount, or dosage, as others. For patients with a bilateral lead configuration, the rate at which stimulation therapy is delivered to the left and right hemispheres can be uneven, as well

The mean, median, 25th and 75th percentiles, and range for accuracy, sensitivity, specificity, had a large spread with an interquartile range of >20% for all metrics (Fig. 4a). Latency had a tighter distribution but still with a large range (−1.1 s–6.1 s). The mean total number of stimulations at 8.5 months post-implant was 355,681 μC/cm^2^ (median 218,710 μC/cm^2^, interquartile range 165,976–510,244 μC/cm^2^, range 73,556–932,899 μC/cm^2^). The histogram revealed a difference in the rate of stimulation therapy between patients, with some patients receiving greater than 10 times the charge amount, or dosage, as others (Fig. 4b). In half of the patients with a bilateral lead configuration, there was a difference in the rate at which stimulation therapy was delivered to the left and right hemispheres of ≥10%, as well.

### Clinical Results

The mean score for patient compliance to using the RNS magnet to mark a seizure event was 2 (Almost never–Half of the time; median 1, interquartile range 1–4, range 1–5), and compliance with maintaining a seizure diary was 3 (Half of the time; median 2, interquartile range 1–3.75, range 1–5). One patient’s primary caretaker passed away and PIES could not be administered. Another patient moved away and could not be reached. A third patient does not currently upload device recordings, due to not having had any clinical seizures since the device was implanted.

The mean patient reported seizure frequency was 59.5 per month (median 30.4, interquartile range 10.3–45.1, range 0–327.4), prior to RNS implantation. The mean reduction in patient reported seizure frequency was 40.1% (median 66.7%, interquartile range 0–96.2%, range − 11.4–100%) with an absolute reduction of 10.9 seizures per month. The mean reduction in seizure duration was 35.8% (median 8.7%, interquartile range 0–82.0%, range − 9.1–100%), and the mean reduction in severity score was 31.6% (median 26.3%, interquartile range 1.7–80.8%, range 0–100%), with an absolute reduction of 1.9 points. The mean time to follow-up was 21.5 months (Table 1; median 22.3, interquartile range 8.8–33.3, range 5.8–36.5). An Engel score of III or better was achieved in 7 patients (58.3%). Pearson correlation showed a trend towards positive correlation between the number of patient reported seizures and corresponding EIPs over a one month period (r = 0.61, *p* = 0.06).

**Table 1:** Patient reported clinical outcomes. Clinical outcomes were measured using a custom seizure questionnaire, the Personal Impact of Epilepsy Scale (PIES) tool developed by Fisher, et al*,* and Engel classification. Both the percent and absolute changes are reported. Better outcomes are indicated by negative numbers for the custom seizure questionnaire and positive number for PIES. Lead locations categorized as cortical dysplasia (CD), neocortical (NC), or mesial temporal (MT).

Patient (lead loc.)	Implant (months)	Seizure Change % (Abs)			PIES Change % (Abs)					Engel Class
		Frequency (per month)	Duration (seconds)	Severity (points)	Sec. A (SEZ)	Sec. B (MED)	Sec. C (COM)	QOL	Overall	
RNS1090 (CD)	5.6	−98.3% (−45.2)	0%	−33.3% (−2)	+1.7% (+1)	+7.3% (+3)	0%	0%	+2.6% (+4)	IIB
RNS1556 (CD)	5.3	0%	0%	−4.3% (−1)	−15.8% (−9)	0%	0%	0%	−5.0% (−9)	IIB
RNS1836 (CD)	19.3	−90.0% (−39.1)	−92.7% (−38)	−81.8% (−9)	+13.3% (+8)	+1.9% (+1)	+6.7% (+2)	+60.0% (+3)	+7.7% (+11)	IIIA
RNS9536 (CD)	33.6	−66.7% (−34.7)	+100% (+20)	+11.1% (+1)	+15.9% (+4)	+6.9% (+4)	0%	0%	+6.9% (+15)	IIIA
RNS1597 (MT)	20.5	0%	0%	−26.3% (−5)	–	–	–	–	–	IVA
RNS1529 (MT)	23.9	0%	0%	0%	0%	0%	−2.7% (−1)	−33.3% (−1)	−0.9% (−1)	IVB
RNS1534 (MT)	10.8	0%	0%	0%	0%	0%	0%	0%	0%	IVB
RNS2227 (MT)	–	–	–	–	–	–	–	–	–	IVC
RNS1603 (NC)	27.4	−100% (−6.5)	−100% (−10)	−100% (−3)	–	−8.0% (−4)	+9.3% (+7)	+14.3% (+1)	–	IB
RNS4098 (NC)	31.8	−100% (−1)	−100% (−1800)	−100% (−5)	+31.0% (+18)	+5.7% (+3)	+53.6% (+30)	+66.7% (+4)	+30.5% (+51)	IB
RNS8076 (NC)	–	–	–	–	–	–	–	–	–	IIA
RNS1440 (NC)	31.8	0%	0%	0%	0%	0%	0%	0%	0%	IVB

## Discussion

We established an analysis pipeline to quantify the extent of bias in electrophysiological event data reported from the RNS System. We found significant bias in how events are reported, secondary to data storage limitations on the device. Our findings demonstrate that standard analysis methods available via the PDMS to understand device behavior can provide misleading results. For this reason, we developed a weighted-means methodology to extrapolate device behavior and partially compensate for data incompleteness and bias.

### Logging Incompleteness and Bias

While it is understood that the RNS System has limited storage capacity, the degree to which its recordings are incomplete and the potential impact on clinical evaluation of recorded events previously has not been shown. This incompleteness can be due to overload of detected events (either true interictal activity or due overly sensitive detection settings), frequency of patient interrogations, insufficient uploads to the laptop, configuration of which recorded events to store, and when the event occurred relative to device interrogation, as only the most recent recordings are saved. The RNS System overwrites the oldest ECoG recordings once the storage limit is reached, which creates a temporally biased sample. In addition, selection bias is introduced when storage slots are allocated to particular triggers, such as magnet swipes, scheduled recordings, *Pattern A*, and *Pattern B*. Despite recent FDA approval of the second generation RNS System, which has double the storage capacity compared to the current generation, these biases will persist.

### Device Behavior Reliability and Heterogeneity

While standard detector performance often reflected the general nature of weighted detector performance, significant differences in magnitude of the detection values were observed. Interpretation of the Bland–Altman plots revealed non-equivalence in all calculations, indicating that the ECoG reports presented on the PDMS represent a significantly biased sample of overall RNS System activity. Further, weighted means were not consistently higher or lower than standard means, demonstrating that general inferences about the direction or magnitude of bias cannot be made. Consequently, observations made solely using ECoG reports on the PDMS can mislead a clinician to unnecessarily modify a well-performing detector or continue a poorly performing detector. It is crucial to appreciate that the ECoG recordings may represent only a small and temporally biased view of what the RNS System is actually detecting and stimulating.

Significant variability in detection accuracy was also observed, where the percentages of types of neurophysiological events being stimulated varied between patients. Likewise, we observed extensive variability in the rate of stimulation, with some patients receiving stimulation at greater than 10 times the frequency of other patients. This heterogeneity with regard to both what is being stimulated and how frequently the stimulation is occurring should be considered when adjusting detection and stimulation parameters, particularly for patients who do not achieve the reported average seizure reduction rates. Additionally, the challenge of adjusting detection thresholds is made more complex by the fact that seizure networks are evolving both via natural history of disease and by modulatory effects of stimulation (Kokkinos et al. 2019). As a result, there is an expected need to regularly optimize detection and stimulation based on monitoring of the closed-loop system.

The finding of a negative detector latency in three of 71 programming epochs is unexpected as it indicates that detection and stimulation immediately preceded the EIP onset. One potential explanation is that false positive stimulation may inadvertently precipitate an EIP event. Such an occurrence does not necessarily correlate to a clinical seizure, and preemptive triggering of a subclinical event could be one of the mechanisms by which the device therapeutically changes seizure dynamics.

### Clinical Response

Approximately half of our patients demonstrated a good clinical response to the RNS System at ≥20 months implanted. This finding further corroborates the RNS clinical trial results of a 50% responder rate at 2 years. Changes in seizure frequency, duration, and severity may reflect the modulatory effects of the RNS System, and these quantifiable endpoints should be monitored to evaluate treatment efficacy. It may be possible to detect these changes using data captured by the RNS System to extrapolate the frequency, duration, and severity of EIPs. For example, patient reported seizure frequency correlated strongly with EIPs (despite a borderline *p* value), further indicating that the latter may be a useful metric for evaluating patient response.

## Limitations

This study was undertaken to address the limitations of working with 90-s snippets of chronic data. While we believe our work shows it is possible to improve upon current methods, we acknowledge it is not possible to fully compensate for missing data. The RNS System’s ability to perform extended recordings is extremely limited, as the propriety wand must be continually pressed to the scalp overlying the device. Because continuous monitoring is not possible, the potential to miss EIPs (false negatives versus true negatives) is inherent due to reliance on detector performance, so we cannot be certain EIPs, and by inference clinical seizures, are not missed. In lieu of continuous recording, we used scheduled recordings to gain insight into brain activity that does not trigger the detector. In the future, physicians may consider continuous scalp EEG post-implantation to augment weighted calculations with a more complete neurophysiologic record.

The PDMS does not display raw data but rather is an abstraction layer that transforms the raw data into something readable by a clinician. As a result, there are sometimes inconsistencies and inaccuracies in what is reported on the PDMS. For example, a magnet swipe may be recorded during an ECoG episode but not displayed in the *Event List*. In another scenario, programming changes made to RNS System may be missing from the *Programming Epochs* tab if they are not properly uploaded from the programming laptop.

The number of patients in this cohort limits statistical analysis. However, the primary focus of this study is to show that current methods for evaluating device behavior are biased and potentially misleading. A larger cohort will be necessary to address the question of how device behavior correlates with closed-loop therapy outcomes. Other potential confounders in correlating RNS System detector performance and stimulation settings with outcomes are changes to the patient’s ASD regimen, heterogeneity of seizure foci location and etiology, and the effect of multidien rhythms in EIP activity on the temporal bias of ECoG recordings (Baud et al. 2018).

## Conclusions

In order to establish a more sophisticated knowledge base from which to ground RNS System programming, we undertook a detailed evaluation of the device capabilities and then built a novel data acquisition and analysis platform to aid in interpreting clinical and ECoG results in the context of the vast parameter space that exists for recording and stimulating. Activity logged by the RNS System is biased and incomplete but can be used, in conjunction with manual review of stored ECoG data, to extrapolate metrics for understanding device behavior. These extrapolated metrics can be unpredictably and significantly different from those derived from currently available metrics on the PDMS. This new framework is useful as an endpoint for device behavior, especially in the context of monitoring patient response to parameter changes, thereby providing basis for adjusting detection and stimulation settings.

### Information Sharing Statement

The MS SQL Server database DDL, query statements used to process and analyze data for this manuscript, as well as MATLAB 2017a scripts used for analysis are available to the community at https://github.com/Brain-Modulation-Lab/rational-approach. For assistance with obtaining RNS System generated data, or loading data into the DDL, please feel free to contact us.

## Electronic supplementary material

ESM 1 (PDF 370 kb)

ESM 2 (PDF 189 kb)

ESM 3 (PDF 79 kb)

ESM 4 (PDF 124 kb)

ESM 5 (PDF 81 kb)

ESM 6 (PDF 88 kb)

ESM 7 (PDF 151 kb)
