# Supplementary material for: A Rational Approach to Understanding and Evaluating Responsive Neurostimulation
Source: Neuroinformatics. 2020 Jan 9;18(3):365–75. doi: 10.1007/s12021-019-09446-7 (PMC7338816; doi:10.1007/s12021-019-09446-7)
Supplement: Supplementary file 1 — (PDF 370 kb) [file 12021_2019_9446_MOESM1_ESM.pdf]

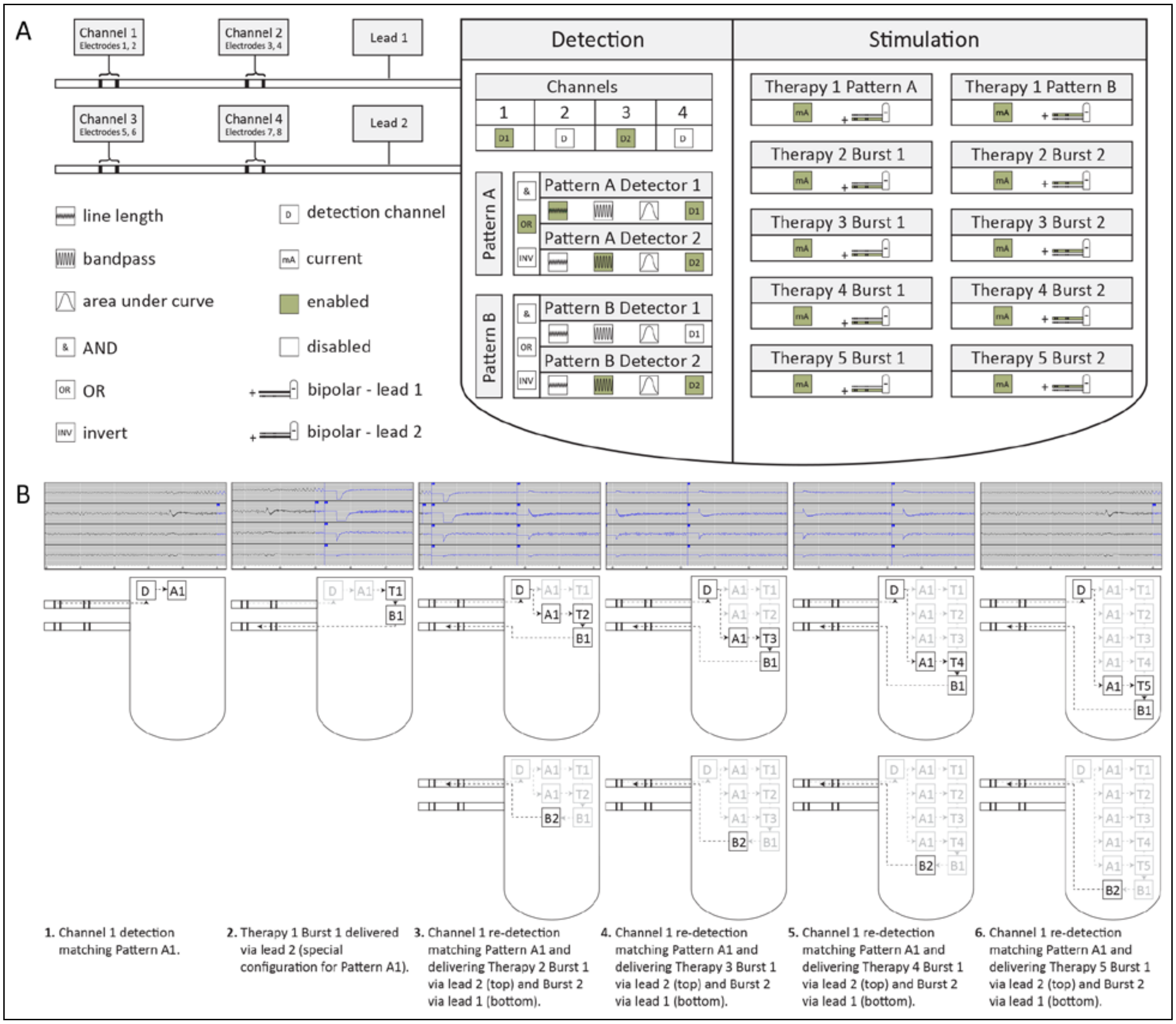

Supporting Figure 1. (**A**) **Simplified representation of RNS System detection and stimulation parameters with sample configuration.** Icons represent configurable groups of configurable settings. Highlighted icons (green) represent a sample of enabled settings. ***Hardware***. Two depth electrodes (leads 1 and 2) are connected to a programmable processor. ***Detection***. Detection is enabled for channels 1 and 2. *Pattern A1* is enabled with a line length detector to trigger when a detection occurs on channel 1. *Pattern A2* is enabled with a bandpass detector to trigger when a detection occurs on channel 2. *Pattern A* triggers when *Pattern A Detector 1* or *Pattern A Detector 2* trigger. *Pattern B2* is enabled with a bandpass detector to trigger when a detection occurs on either channel 2. *Pattern B1* is disabled. ***Stimulation****.* Therapy 1 only is configured with different response for *Pattern A* versus *Pattern B*. The montage is configured in bipolar fashion to deliver subsequent therapy in bursts that alternate between lead 1 and lead 2. (**B**) **Closed-loop sequence of events.** This sequence of events shows how the sample configuration (see Figure 1) responds to monitored brain activity. Each ECoG snippet (top) represents activity that corresponds with a schematic (middle) and description (below). Detection (D) occurs, followed by a therapy (T) comprised of up to two bursts (B). Each subsequent therapy is contingent upon re-detection, and represents a closed-loop; two bursts may occur for a single re-detection.
