# Supplementary material for: A Rational Approach to Understanding and Evaluating Responsive Neurostimulation
Source: Neuroinformatics. 2020 Jan 9;18(3):365–75. doi: 10.1007/s12021-019-09446-7 (PMC7338816; doi:10.1007/s12021-019-09446-7)
Supplement: Supplementary file 2 — (PDF 189 kb) [file 12021_2019_9446_MOESM2_ESM.pdf]

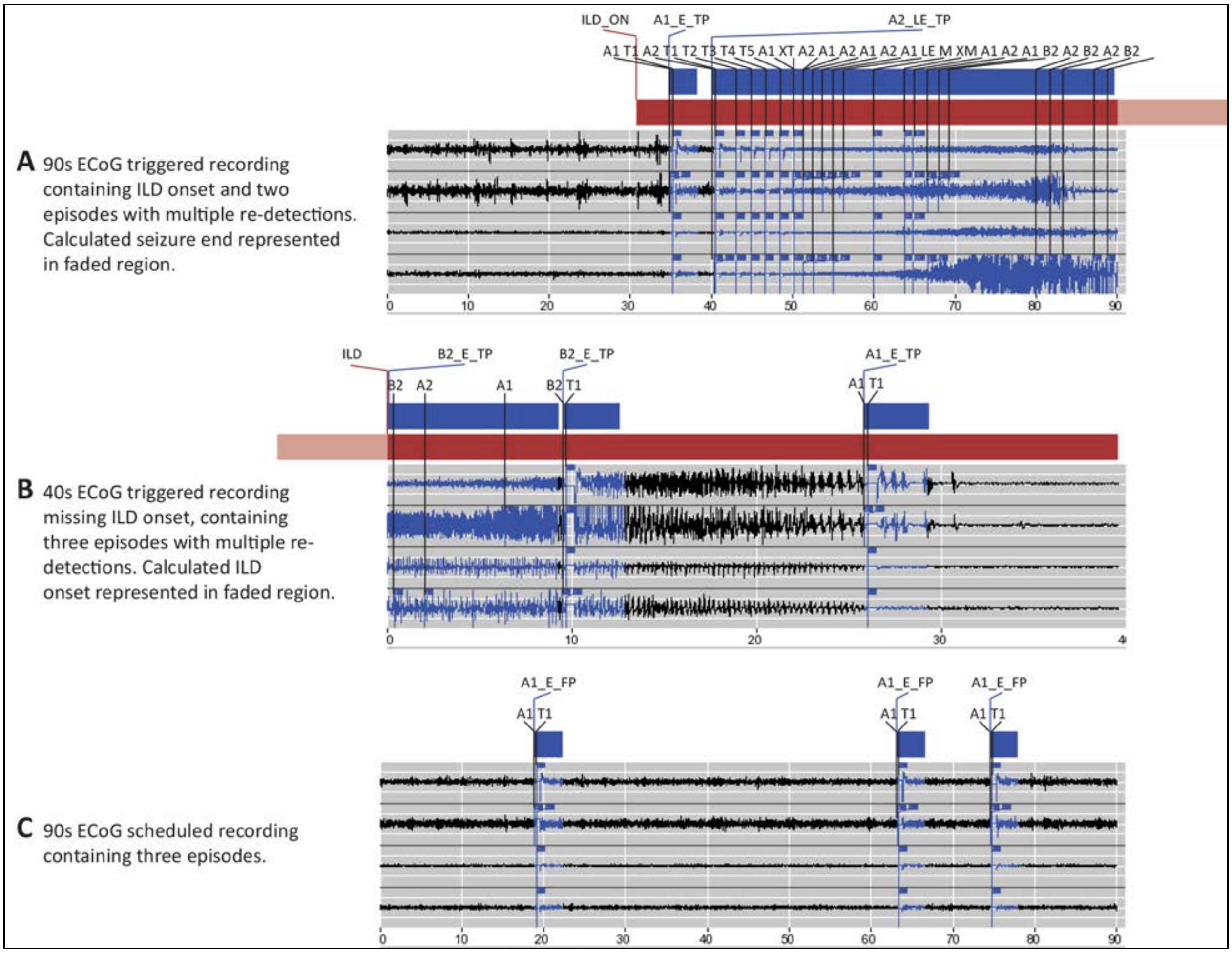

Supporting Figure 2. Labeled ECoG recordings demonstrating three common scenarios for classifying episodes. *ILD = ictal-like discharge with missing onset; ILD_ON = ictal discharge onset; A1_E_TP = Pattern A1 episode, true positive; A1_LE_TP = Pattern A1 long episode, true positive.*
