# Supplementary material for: A Rational Approach to Understanding and Evaluating Responsive Neurostimulation
Source: Neuroinformatics. 2020 Jan 9;18(3):365–75. doi: 10.1007/s12021-019-09446-7 (PMC7338816; doi:10.1007/s12021-019-09446-7)
Supplement: Supplementary file 3 — (PDF 79 kb) [file 12021_2019_9446_MOESM3_ESM.pdf]

1. **Seizure Types and Outcomes**

Seizure Type 1 Description:

Frequency	Duration	Severity (1 = Not severe, 5 = Very severe)	Consciousness (Y/N)

Seizure Type 2 Description:

Frequency	Duration	Severity (1 = Not severe, 5 = Very severe)	Consciousness (Y/N)

Seizure Type 3 Description:

Frequency	Duration	Severity (1 = Not severe, 5 = Very severe)	Consciousness (Y/N)

Seizure Type 4 Description:

Frequency	Duration	Severity (1 = Not severe, 5 = Very severe)	Consciousness (Y/N)

Seizure Type 5 Description:

Frequency	Duration	Severity (1 = Not severe, 5 = Very severe)	Consciousness (Y/N)

2. **I magnet swipe to mark a seizure:**

1	2	3	4	5
Almost never		Half of the time		Almost always

3. **I record seizure activity in the online diary:**

1	2	3	4	5
Almost never		Half of the time		Almost always

Supporting Figure 3. Custom questionnaire regarding seizure frequency, duration, severity, and consciousness. This survey, and the complementary PIES survey tool, are administered at each RNS patient visit.
