# Supplementary material for: A Rational Approach to Understanding and Evaluating Responsive Neurostimulation
Source: Neuroinformatics. 2020 Jan 9;18(3):365–75. doi: 10.1007/s12021-019-09446-7 (PMC7338816; doi:10.1007/s12021-019-09446-7)
Supplement: Supplementary file 4 — (PDF 124 kb) [file 12021_2019_9446_MOESM4_ESM.pdf]

**Anode**	The positive pole of an electrode selected for stimulation.
**Area detection tool**	The area detection tool measures the area under the curve of an ECoG signal.
**Bandpass detection tool**	The bandpass detection tool measures the approximate frequency of the signal based on the duration and amplitude of waveform segments.
**Burst**	An individually programmed group of one to many pulse(s) of current delivered by the neurostimulator.
**Burst duration**	The amount of time during which a burst will be delivered by the neurostimulator.
**Burst 1**	The first burst of stimulation delivered within an individual responsive therapy. This burst may be delivered asynchronously or synchronously.
**Burst 2**	The second burst of stimulation delivered within an individual responsive therapy. This burst may be programmed ON or OFF for each therapy and will always be delivered asynchronously following Burst 1 of the therapy if programmed ON.
**Calculated charge density**	The charge density of a selected and/or programmed stimulation is calculated by the programmer. Charge densities greater than 25 $\mu C/cm^2$/phase are not allowed to be selected or programmed into the neurostimulator.
**Cathode**	The negative pole of an electrode selected for stimulation.
**Configure detection**	Analysis of detection settings using the programmer detection tools and retrieved neurostimulator ECoG records.
**Cortical Strip Lead**	The cortical strip lead is intended for subdural implant to provide an interface through which electrical activity of the brain can be monitored and electrical stimulation may be delivered.
**Current**	The amplitude of current that will be delivered in each phase of a current pulse.
**Depth Lead**	The depth lead is intended for implant into deep brain structures to provide an interface through which electrical activity of the brain can be monitored and electrical stimulation may be delivered.
**Detector channel**	The amplifier channel of electrodes selected to detect ECoG activity.
**Detection settings**	Settings that combine patterns and detection tool settings in order to detect activity.
**Detection tools**	There are three (3) types of detection tools selectable for Pattern A or Pattern B event detectors. The detection tools are: the bandpass tool, line length tool, and area tool.
**ECoG. Electrocorticogram**	Electrical activity derived directly from the cerebral cortex. Also used to describe the neurostimulator or programmer stored record of this activity (e.g. “ECoG record”).
**ECoG activity**	ECoG activity refers to activity which is detected by the neurostimulator using one of the event detectors.
**ECoG Length**	The amount of time stored for a programmer commanded ECoG record.
**ECoG storage trigger**	The type of neurostimulator activity selected by the user to result in the storage of an ECoG record if the activity occurs.
**Episode duration**	The time elapsing between the point at which the neurostimulator detects Pattern A or Pattern B to the point at which post-episode baseline activity is detected.

**Event**	Activity detected by the neurostimulator. Events are considered any of the following: long episodes, pattern A, pattern B, responsive therapy, noise or saturation.
**Frequency**	The frequency is the rate at which pulses are delivered which is expressed in Hz (pulses/second).
**Impedance measurement**	The opposition of current flow between electrodes as determined by measuring the voltage (Ohms) resulting from the delivery of a current pulse. A current pulse must be applied to the patient in order to obtain this measurement.
**Implant configuration**	Information which describes the port(s) to which the proximal end of the lead(s) are connected.
**Interrogate**	The act of retrieving stored data and settings from the neurostimulator using the programmer.
**Line length detection tool**	The line length detection tool performs an estimate of the length dimension of a signal related to the complexity of a signal.
**Long episode**	Initiate ECoG storage when a detected episode continues beyond a preset duration.
**Magnet**	The magnet suppresses RNS® System therapy as long as the magnet is in position. Placing the magnet over the neurostimulator also triggers a diagnostic, and can also be programmed to trigger ECoG storage.
**Montage**	Collective term used to describe the assignment of electrode labels and amplifier gain settings to the amplifier channels.
**Noise**	1. An event detector that identifies 60 Hz noise from selected detect channels. 2. Environmental interference in the telemetry between the neurostimulator and wand/programmer.
**Overwritten**	Once the memory for ECoG or diagnostics is full, the neurostimulator will store new data by overwriting the oldest ECoG records or diagnostics.
**Patient Data Management System (PDMS)**	The PDMS is a secure web site that provides a means to review information that has been up-loaded by the programmer.
**Patterns**	Patterns (pattern A and pattern B) use a collection of detection tools to classify electrographic activity.
**Post-episode monitoring interval**	Responsive therapies will be DISABLED for this period of time after detecting the end of an episode.
**Post-episode therapy**	Therapy that is delivered following an episode containing responsive therapy. Post-episode therapy will only be delivered if the setting is programmed as ENABLED.
**Post-trigger ECoG storage**	Neurostimulator setting which indicates the duration of ECoG data that will be stored in the neurostimulator after an ECoG triggering event.
**Pre-trigger ECoG storage**	Neurostimulator setting which indicates the duration of ECoG data that will be stored in the neurostimulator prior to an ECoG triggering event.
**Programmer**	The programmer is a laptop personal computer that runs software developed by NeuroPace to communicate with the neurostimulator, to download selected settings into the neurostimulator, and to upload information from the neurostimulator. The programmer communicates with the neurostimulator via the wand. The programmer also communicates with the PDMS via an internet connection.

**Programming**	Using the programmer to program settings into the neurostimulator.
**Pulse**	A biphasic waveform of electrical stimulation delivered by the neurostimulator.
**Pulse count**	The pulse count is the number of times a pulse will be delivered in a burst. This count is calculated and displayed on the programmer for each selected therapy.
**Pulse interval type**	For responsive therapy bursts, the pulse interval type can be selected as adaptive or fixed.
**Pulse to pulse (p-p) interval**	The duration between the start of one stimulation pulse and the start of a subsequent pulse.
**Pulse-width per phase (PW)**	Duration of a single phase within a biphasic pulse, measured in milliseconds.
**Recovery period**	Period of time after the end of noise detection during which the neurostimulator attempts to determine post-episode baseline activity.
**Reset**	When the neurostimulator is reset the following will occur: detection and all therapies will be disabled, no ECoGs will be stored, and no diagnostics will be stored (however events prior to reset will be saved). If a neurostimulator reset occurs, the user should note the cause of the reset and contact NeuroPace.
**Responsive therapy**	Electrical stimulation output to cortical tissue by the neurostimulator in response to pattern A or pattern B detection.
**RNS® Neurostimulator**	An implantable, battery powered, microprocessor controlled device that can amplify and analyze the patient's electrocorticographic activity, detect activity from intracranial electrodes and deliver a short train of current pulses to the brain to attempt to interrupt the detected activity.
**Saturation event detector**	Saturation occurs when an input signal exceeds the dynamic range of a particular channel. The neurostimulator incorporates a saturation detector to reduce occurrences of detecting saturation as a neurological event.
**Scheduled ECOG storage**	A selectable neurostimulator function that causes the neurostimulator to store an ECoG at the desired time(s) of day.
**Stim pathway**	Collective term describing the anodes (+) and cathodes (-) selected for a burst.
**Storage start time**	Scheduled ECoG storage setting controlling the time(s) at which ECoG storage will be triggered.
**Summary**	Programmer interactive display of a record of the neurostimulator activity occurring since the last interrogation.
**Synchronize data**	The action of transferring data (via the internet) to the PDMS.
**Technical Mode**	Option to configure detection using all detection parameters.
**Telemetry**	Communication between the neurostimulator and the programmer.
**Therapy limit per day**	Responsive therapy delivery will be DISABLED for the remainder of the day after the programmed number of responsive therapies has been delivered.
**Therapy sequence**	Responsive therapy is delivered as a therapy sequence of up to 5 individually configured sequential therapies (electrical stimulation) in response to each detected episode.

Supporting Table 1. Glossary of RNS System and related terminology. RNS System definitions reprinted from RNS System Manual with permission.
