# Supplementary material for: A Rational Approach to Understanding and Evaluating Responsive Neurostimulation
Source: Neuroinformatics. 2020 Jan 9;18(3):365–75. doi: 10.1007/s12021-019-09446-7 (PMC7338816; doi:10.1007/s12021-019-09446-7)
Supplement: Supplementary file 5 — (PDF 81 kb) [file 12021_2019_9446_MOESM5_ESM.pdf]

| | | |
|---|---|---|
| **Total Patients** | | 12 |
| | Male | 4 |
| | Female | 8 |
| **Mean age at implant** | | 35.6 ± 11.9 years |
| **Mean age of seizure onset** | | 17.1 ± 11.4 years |
| **Mean years of seizures** | | 18.5 ± 10.4 years |
| **Mean failed anti-seizure drugs** | | 2.8 ± 0.8 drugs |
| **Mean months implanted with RNS** | | 21.5 ± 10.4 months |
| **Etiologies** | Structural | 8 |
| | Immune | 1 |
| | Genetic | 3 |
| | Unknown | 3 |
| **Implant locations** | Mesial temporal | 7 |
| | Neocortical | 5 |
| **Mean seizure tracking compliance** | Seizure Diary | 2.6 ± 1.7 out of 5 |
| | Magnet | 3.0 ± 1.7 out of 5 |

**Supporting Table 2. Summary demographic data describing the RNS System-implanted patient population at UPMC.** Etiologies are classified according to the ILAE 2017 scheme, and a single patient may have multiple etiologies. Mean seizure tracking compliance was measured on a scale of 1–Almost never to 5–Almost always (see **Supplemental 4**).
