# Supplementary material for: A Rational Approach to Understanding and Evaluating Responsive Neurostimulation
Source: Neuroinformatics. 2020 Jan 9;18(3):365–75. doi: 10.1007/s12021-019-09446-7 (PMC7338816; doi:10.1007/s12021-019-09446-7)
Supplement: Supplementary file 6 — (PDF 88 kb) [file 12021_2019_9446_MOESM6_ESM.pdf]

Patient	No.	Class 1	Class 2	Class 3	Class 4	Class 5
RNS1090	1	focal onset	impaired awareness	non-motor onset	cognitive	n/a
RNS1440	1	focal onset	impaired awareness	non-motor onset	sensory	n/a
RNS1529	1	focal onset	impaired awareness	non-motor onset	sensory	bilat. tonic-clonic
RNS1534	1	focal onset	impaired awareness	non-motor onset	sensory	n/a
RNS1556	1	focal onset	aware	non-motor onset	sensory	n/a
	2	focal onset	impaired awareness	non-motor onset	cognitive	bilat. tonic-clonic
RNS1597	1	focal onset	impaired awareness	motor onset	automatisms	bilat. tonic-clonic
	2	focal onset	impaired awareness	non-motor onset	cognitive	bilat. tonic-clonic
RNS1603	1	focal onset	aware	motor onset	tonic	n/a
	2	focal onset	impaired awareness	motor onset	tonic	bilat. tonic-clonic
RNS1836	1	focal onset	aware	motor onset	automatisms	n/a
	2	focal onset	impaired awareness	non-motor onset	autonomic	bilat. tonic-clonic
RNS2227	1	focal onset	impaired awareness	non-motor onset	cognitive	bilat. tonic-clonic
	2	focal onset	impaired awareness	non-motor onset	autonomic	bilat. tonic-clonic
RNS4098	1	focal onset	aware	non-motor onset	cognitive	n/a
	2	focal onset	aware	non-motor onset	sensory	n/a
RNS8076	1	focal onset	aware	non-motor onset	myoclonic	bilat. tonic-clonic
	2	focal onset	impaired awareness	motor onset	cognitive	n/a
RNS9536	1	focal onset	impaired awareness	motor onset	hyperkinetic	bilat. tonic-clonic
	2	focal onset	impaired awareness	non-motor onset	emotional	n/a
	3	focal onset	impaired awareness	non-motor onset	sensory	n/a

Supporting Table 3. Seizure classification per patient. Unique seizure types per patient were classified according to the ILAE 2017 schema.
