# Supplementary material for: A Rational Approach to Understanding and Evaluating Responsive Neurostimulation
Source: Neuroinformatics. 2020 Jan 9;18(3):365–75. doi: 10.1007/s12021-019-09446-7 (PMC7338816; doi:10.1007/s12021-019-09446-7)
Supplement: Supplementary file 7 — (PDF 151 kb) [file 12021_2019_9446_MOESM7_ESM.pdf]

Patient		Programming Epoch									
		1	2	3	4	5	6	7	8	9	10
RNS1090	STA	61.6%*	95.3%	97.4%							
	WTA	72.2%*	99.1%	99.0%							
	LAT	–	-0.596s	2.071s							
	SEN	40.0%*	95.0%	41.3%							
	SPE	64.0%*	95.3%	98.4%							
RNS1440	STA	98.1%	96.1%	63.8%	49.1%	45.0%	99.0%	99.0%			
	WTA	98.5%	95.8%	83.7%	70.1%	74.0%	98.8%	98.4%			
	LAT	4.866s	0.815s	0.174s	0.060s	0.087s	0.413s	1.711s			
	SEN	7.3%	71.3%	93.6%	96.8%	99.0%	90.5%	70.1%			
	SPE	100%	98.0%	61.1%	36.2%	38.0%	100%	100%			
RNS1529	STA	98.8%*	83.8%	86.0%	94.0%	92.0%	80.0%	83.0%*			
	WTA	97.9%*	91.1%	92.2%	92.9%	89.3%	84.4%	84.2%*			
	LAT	–	0.199s	3.755s	2.667s	2.959s	2.872s	2.644s*			
	SEN	71.3%*	42.1%	87.2%	89.7%	98.9%	87.6%	86.0%*			
	SPE	99.9%*	92.3%	86.2%	95.0%	90.0%	78.1%	82.0%*			
RNS1534	STA	100%*	78.4%	75.5%	80.3%	80.2%					
	WTA	99.9%*	89.9%	88.9%	90.3%	89.3%					
	LAT	4.241s*	–	1.939s	1.337s	3.046s					
	SEN	93.3%*	51.5%	40.4%	48.1%	38.6%					
	SPE	100%*	80.1%	77.1%	81.9%	82.1%					
RNS1556	STA	100%*	99.5%								
	WTA	99.8%*	99.6%								
	LAT	–	1.599s								
	SEN	79.6%*	33.0%								
	SPE	100%*	99.5%								
RNS1597	STA	99.0%	86.1%	63.2%	59.2%	73.7%					
	WTA	98.8%	92.4%	69.7%	64.5%	82.2%					
	LAT	4.228s	3.244s	2.226s	1.076s	2.754s					
	SEN	94.3%	87.5%	74.2%	66.4%	74.9%					
	SPE	99.5%	86.2%	61.4%	55.9%	74.0%					
RNS1603	STA	97.1%	95.2%	94.6%	84.8%	93.3%	88.4%	89.7%			
	WTA	99.2%	95.9%	95.4%	87.6%	95.6%	92.0%	93.3%			
	LAT	5.505s	6.078s	3.567s	3.006s	1.714s	-1.073s	-0.278s			
	SEN	3.5%	59.8%	40.0%	50.6%	48.4%	52.6%	51.9%			
	SPE	97.1%	95.2%	94.6%	85.3%	93.9%	90.4%	92.4%			
RNS1836	STA	88.1%*	80.0%	60.9%	64.7%						
	WTA	81.4%*	82.9%	72.8%	68.0%						
	LAT	2.664s*	3.123s	2.268s	3.141s						
	SEN	73.3%*	50.7%	59.6%	59.4%						
	SPE	96.6%*	92.1%	61.7%	71.5%						
RNS2227	STA	100%	97.0%	92.7%	96.5%						
	WTA	99.9%	98.2%	95.0%	97.8%						
	LAT	2.477s	1.315s	1.888s	1.752s						
	SEN	36.6%	87.9%	81.8%	71.1%						

	SPE	100%	97.4%	93.3%	97.0%						
RNS4098	STA	100%	97.8%	95.0%	99.5%	93.0%	80.1%	69.1%	45.7%	56.6%	64.5%
	WTA	99.4%	99.0%	95.4%	93.8%	84.8%	66.9%	66.8%	63.0%	64.6%	69.0%
	LAT	–	–	–	2.085s	1.000s	0.612s	0.664s	0.541s	0.854s	0.839s
	SEN	0%	0%	0%	96.4%	98.6%	99.8%	99.7%	97.7%	98.9%	99.6%
	SPE	100%	97.8%	100%	100%	91.2%	62.3%	52.3%	34.5%	42.5%	54.4%
RNS8076	STA	100%	98.5%	99.1%	99.0%	100%					
	WTA	99.9%	99.4%	99.7%	99.6%	99.9%					
	LAT	0.400s	0.458s	0.217s	0.348s	0.380s					
	SEN	20.2%	29.0%	7.0%	37.3%	98.6%					
	SPE	100%	100%	100%	100%	100%					
RNS9536	STA	100%	100%	98.1%*	80.0%	76.0%	84.5%	82.3%	67.5%	67.5%	75.4%
	WTA	100%	99.8%	99.3%*	96.2%	95.6%	97.0%	96.5%	93.6%	93.4%	94.8%
	LAT	–	3.375s	1.500s*	1.885s	1.260s	1.688s	2.519s	2.219s	1.700s	3.060s
	SEN	86.9%	54.4%	99.9%*	100%	83.7%	81.8%	61.6%	76.8%	81.7%	84.3%
	SPE	100%	100%	98.1%*	79.1%	76.0%	84.5%	83.3%	66.5%	66.5%	75.4%

Supporting Table 4. Standard accuracy (STA), weighted accuracy (WTA), latency (LAT), sensitivity (SEN), and specificity (SPE) per programming epoch per patient. Calculations reflecting detector performance, as well as the difference between standard (raw) and weighted (extrapolated) accuracy, are reported for each programming epoch in which changes to the detector settings were made. Calculations for which no ECoG recordings were available for one or more *Patterns* are marked with an asterisk (*).
